# Electromembrane Extraction of Highly Polar Compounds: Analysis of Cardiovascular Biomarkers in Plasma

**DOI:** 10.3390/metabo10010004

**Published:** 2019-12-18

**Authors:** Nicolas Drouin, Tim Kloots, Julie Schappler, Serge Rudaz, Isabelle Kohler, Amy Harms, Petrus Wilhelmus Lindenburg, Thomas Hankemeier

**Affiliations:** 1 Institute of Pharmaceutical Sciences of Western Switzerland (ISPSO), University Medical Centre, 1206 Geneva, Switzerland; n.f.p.drouin@lacdr.leidenuniv.nl (N.D.); julie.schappler@icloud.com (J.S.); serge.rudaz@unige.ch (S.R.); 2 Division of Systems Biomedicine and Pharmacology, Leiden Academic Center for Drug Research, Leiden University, Einsteinweg 55, 2333 CC Leiden, The Netherlands; t.n.kloots@lacdr.leidenuniv.nl (T.K.); i.kohler@lacdr.leidenuniv.nl (I.K.); a.c.harms@lacdr.leidenuniv.nl (A.H.); hankemeier@lacdr.leidenuniv.nl (T.H.); 3 Research Group Metabolomics, Leiden Centre for Applied Bioscience, University of Applied Sciences Leiden, Zernikedreef 11, 2333 CK Leiden, The Netherlands

**Keywords:** electromembrane extraction, cardiovascular disease, multi-segment injection, capillary electrophoresis–mass spectrometry, liquid chromatography–mass spectrometry

## Abstract

Cardiovascular diseases (CVDs) represent a major concern in today’s society, with more than 17.5 million deaths reported annually worldwide. Recently, five metabolites related to the gut metabolism of phospholipids were identified as promising predictive biomarker candidates for CVD. Validation of those biomarker candidates is crucial for applications to the clinic, showing the need for high-throughput analysis of large numbers of samples. These five compounds, trimethylamine N-oxide (TMAO), choline, betaine, l-carnitine, and deoxy-l-carnitine (4-trimethylammoniobutanoic acid), are highly polar compounds and show poor retention on conventional reversed phase chromatography, which can lead to strong matrix effects when using mass spectrometry detection, especially when high-throughput analysis approaches are used with limited separation of analytes from interferences. In order to reduce the potential matrix effects, we propose a novel fast parallel electromembrane extraction (Pa-EME) method for the analysis of these metabolites in plasma samples. The evaluation of Pa-EME parameters was performed using multi segment injection–capillary electrophoresis–mass spectrometry (MSI-CE-MS). Recoveries up to 100% were achieved, with variability as low as 2%. Overall, this study highlights the necessity of protein precipitation prior to EME for the extraction of highly polar compounds. The developed Pa-EME method was evaluated in terms of concentration range and response function, as well as matrix effects using fast-LC-MS/MS. Finally, the developed workflow was compared to conventional sample pre-treatment, i.e., protein precipitation using methanol, and fast-LC-MS/MS. Data show very strong correlations between both workflows, highlighting the great potential of Pa-EME for high-throughput biological applications.

## 1. Introduction

With 17.5 million deaths per year, cardiovascular disease (CVD) represents the leading cause of death worldwide and is thus considered a major public health issue [1]. Gut flora-dependent metabolism has been recently reported as a risk factor for CVD, most notably the metabolic pathway for dietary phosphatidylcholine which includes trimethylamine oxide (TMAO), choline, betaine, l-carnitine, and deoxy-l-carnitine. These metabolites are related to the development of various CVDs including stroke and myocardial dysfunction [2,3,4,5].

Mass spectrometry (MS) coupled with separation techniques is a widely used tool in metabolomics, enabling the identification and quantitation of metabolites via the analysis of thousands of samples. Hydrophilic interaction chromatography (HILIC)-based methods allow for an efficient separation of polar compounds such as the ones involved in the dietary phosphatidylcholine metabolic pathway, but they require long re-equilibration times, resulting in low throughput [6]. Fast chromatography is often preferred in combination with fast sample pre-treatment. However, this approach may lead to significant matrix effects (MEs), i.e., the alteration of the analyte signal due to the presence of co-eluting matrix interference. Deuterated internal standards (dISTDs) can be added to correct for the deleterious effect of ME on quantitation. However, even though quantitative corrections can be applied, ion suppression will still lead to decreased signal intensity and lower sensitivity, showing the need for alternative approaches.

In order to reduce MEs, a sample preparation step is highly recommended. The most common practice in metabolomics is to remove proteins by protein precipitation [7] or ultrafiltration [8]. However, these two approaches cannot separate the compounds of interest from interferences such as salts or phospholipids [9,10]. To do this, more complex sample preparation approaches, e.g., liquid–liquid extraction or reversed phase solid phase extraction are often considered. However, in the present case the metabolites of interest are quaternary ammoniums. Therefore, only weak cation exchanger resins are conceivable. However, this extraction mode is complicated and expensive.

Electromembrane extraction (EME) is a recently developed electro-driven sample preparation method developed for the extraction of charged compounds [11]. In this approach, two compartments, namely, the donor and acceptor compartments, are separated by a supported liquid membrane (SLM) containing an organic solvent. When applying an electric field between the two compartments, the ions present in the donor compartment are selectively extracted to the acceptor compartment [12,13]. With this approach, it is possible to reach very high recovery (up to 100%) and enrichment factor (up to 50-fold) in a few minutes [14,15]. In addition, this method allows for an efficient sample clean-up by separation of metabolites of interest from phospholipids and salts, as well as proteins [16,17,18]. EME has extensively been applied to the extraction of non-polar and moderately polar drugs (1 < logP < 5). Recent work has also demonstrated the great potential of EME for the extraction of highly polar compounds (logP < −1) [19,20,21,22]. TMAO, choline betaine, l-carnitine, and deoxy-l-carnitine, as quaternary ammonium compounds, are excellent candidates for EME due to their permanent positive charge. Indeed, acylcarnitines have been already successfully extracted by electroextraction [23,24,25]. However, the EME set-ups described are not suited for high throughput workflows necessary for large cohort studies.

In this study, we developed and optimized a parallel EME (Pa-EME) extraction method for the these five metabolites from human plasma samples. This approach enabled the extraction from several samples in parallel, dramatically increasing the extraction throughput. A non-polar drug, bupivacaine, was also added to the set of selected compounds to monitor the extraction process. The influence of the applied voltage as well as the composition of the donor compartment were investigated. For method optimization, the extracted compounds were analyzed using a multi segment-capillary electrophoresis-mass spectrometry (MSI-CE-MS) method to both improve the throughput of the analysis and reduce the analytical variability. The optimized method was then combined with fast-liquid chromatography–tandem mass spectrometry (Fast-LC-MS/MS) for further evaluation of response function and dynamic range. ME was evaluated by comparing the developed extraction method to a sample pre-treatment method conventionally used in metabolomics, i.e., protein precipitation using methanol (PP-MeOH) [26,27]. Finally, the developed EME-LC-MS/MS method was applied to the analysis of 40 plasma samples and compared to PP-MeOH to show the strength of the method. This highlighted the relevance of such approaches for large-scale metabolomics studies, where the analysis of highly polar metabolites in complex matrices remains challenging.

## 2. Experimental

### 2.1. Chemical and Reagents

MS-grade acetic acid and sodium hydroxide (≥ 99%) were purchased from VWR International BV (Amsterdam, The Netherlands). MS-grade methanol (MeOH), isopropanol (iPrOH), and acetonitrile (MeCN) were supplied from Actu-All Chemicals (Oss, the Netherlands). Model compounds (purity ≥ 95%) as well as 6.1 N trichloroacetic acid (TCA) and 2-nitrophenyl pentyl ether (NPPE, ≥ 99%) were purchased from Sigma-Aldrich Chemie NV (Zwijndrecht, The Netherlands). Deuterated internal standards (dISTDs), isotopic purity ≥ 99%, were purchased from Cambridge Isotopes Laboratories, Inc. (Tewksbury, MA, USA), except TMAO-d9 (CDM Isotopes, Pointe-Claire, Quebec, Canada). MS-grade formic acid (FA) and 37% (*v*/*v*) hydrochloric acid were obtained from Thermo Fisher Acros Organics (Geel, Belgium)

### 2.2. Preparation of Standard Solutions

Individual stock solutions of both standard and deuterated internal standard (dISTD) were prepared in water/MeCN/FA (94.9:5:0.1, *v/v*) to reach a final concentration of 5 mg/mL. The method development was performed using neat standards of carnitine-d_3_, deoxycarnitine-d_9_, choline-d_4_, betaine-d_9_, TMAO-d_9_, and bupivacaine prepared in 5% MeCN and 0.1% FA at 100 µg/mL. This mixture was diluted in the donor solution to a final concentration of 2.5 µg/mL before use. The acceptor solution consisted of a solution of 1% acetic acid.

In addition, for MeOH for protein precipitation, dISTD solutions were also prepared in MeOH at the following concentrations: 4 µg/mL for carnitine-d3, 0.2 µg/mL for deoxycarnitine-d9, 2 µg/mL for choline-d4, 4 µg/mL for betaine-d9, and 0.3 µg/mL for TMAO-d9.

### 2.3. Plasma Samples

The method development was carried out using citrate plasma samples collected from eight healthy volunteers obtained from Sanquin (Amsterdam, The Netherlands) and pooled together.

The method comparison was performed with 40 plasma samples collected from healthy volunteers from the Growing Old Together (GOTO) study [28].

### 2.4. Sample Preparation: Protein Precipitation

In order to enhance extraction performance of Pa-EME, three different PP methods were investigated during this study, namely, (1) PP using 6.1 N TCA (referred to as PP-TCA), (2) adjusted PP-TCA, and (3) PP using MeOH with a ratio MeOH/sample of 9:1 ratio (referred to as PP-MeOH). The resulting solutions were used as donor solution during the Pa-EME procedure.

The PP-TCA was performed using a solution of 6.1 N TCA with a ratio of 0.05:1 (TCA/sample, *v/v*). Briefly, 25 µL of 6.1 N TCA were added to 475 µL of plasma. After 30 min of agitation at 1400 rpm at 23 °C using a Thermomixer (Vaudaux-Eppendorf AG, Bale, Switzerland), the sample was centrifuged at 15,000 rpm for 15 min at 4 °C. Supernatant was then collected. In order to avoid potential variability, multiple PPs were performed and their supernatants were mixed together prior to further division into aliquots. Aliquots were then kept at −20 °C until analysis. As a donor solution for Pa-EME, the aliquots were then diluted with adequate volume of water to reach an equivalent concentration of 10, 20, and 50% of untreated plasma, respectively.

This PP-TCA method was then adapted for the analysis of the 40 different plasma samples due to the small volume of sample available, i.e., ca. 25 µL. Briefly, 20 µL of plasma were mixed with 20 µL of dISTD solution. Then, 2 µL of 6.1 N TCA were added to obtain a final ratio of TCA/sample of 0.05:1 (*v/v*). After agitation at 1400 rpm for 30 min at 23 °C, 358 µL of water was added prior to centrifugation at 24,400× *g* during 20 min at 4 °C. The supernatant was then collected and stored at −20 °C prior to electroextraction. For EME, 300 µL of the supernatant was used, leading to an equivalent untreated plasma content of 5% in the donor compartment.

The third method, namely, PP-MeOH, was performed using 90 µL of MeOH containing dISTD added to 10 µL of plasma. After vortex agitation for 1 min, samples were centrifuged for 5 min at 18,300× *g*. The supernatant was then collected, leading to a corresponding concentration of 10% of untreated plasma in the analyzed sample.

### 2.5. Pa-EME Set-Up and Procedure

**Figure 1:**
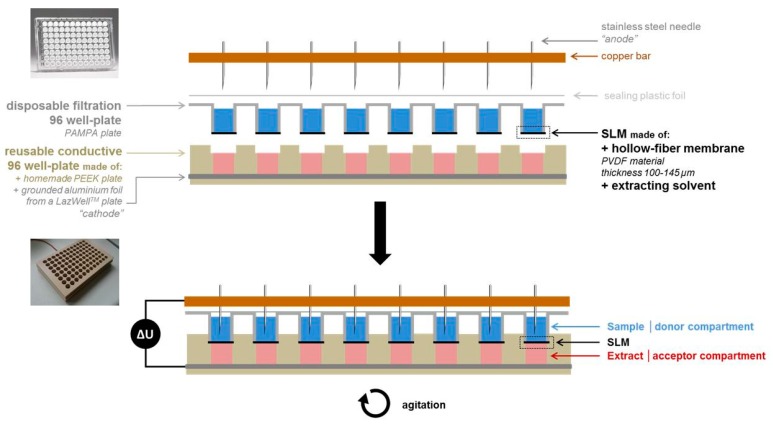
Schematic representation of the parallel electromembrane extraction (Pa-EME) device. Reprinted with permission [29]. SLM: supported liquid membrane; PAMPA: Parallel artificial membrane permeability assay; PVDF: polyvinyldifluoride.

The electroextraction procedure and the Pa-EME device used in this study have been already described elsewhere [28]. Briefly, the Pa-EME device consisted of a donor and an acceptor 96 well-plate, as is illustrated in Figure 1. The acceptor plate consists of a custom made conductive 96 well-plate in polyether ether ketone (PEEK) polymer. Conductivity was ensured by a piece of aluminum foil with a thickness of 0.14 mm placed on the bottom of the well plate. In order to limit carryover and cross contamination, the donor plate used was a disposable MultiScreen-IP Filter Plate of 300 µL with a polyvinyldifluoride (PVDF) membrane with a thickness of 100 to 145 μm and a pore size of 0.45 μm, and purchased from Millipore (Milford, MA, USA).

Impregnation of the PVDF membrane was performed by pipetting 3 µL of 2-nitrophenylpentyl ether (NPPE) on the external face of the PVDF. Then, the excess of SLM is removed by placing the plate on a tissue and by application of a 2.5 psi for 5s in each well using a positive pressure manifold (Biotage, Uppsala, Sweden). This cleaning step was repeated until the tissue appeared dry. The donor compartment was then filled with 300 µL of sample and was sealed using an adhesive sealing film (PCR-TS, Axygen, MA, USA).

After filling of the acceptor plate with 300 µL of acceptor solution made of 1% acetic acid, the donor plate was inserted into the acceptor one and the Pa-EME system was placed on a thermomixer for agitation. The electrode needles were then inserted in the donor compartment. Extraction took place for 15 min at 1400 rpm, with application of a constant voltage or current between the needles and the aluminum foil of the conductive well-plate using a Power Supply ES 0300–0.45 from Delta Elektronica (Zierikzee, the Netherlands). After 15 min, the power supply was turned off, needles were removed from the acceptor plate, and both plates were separated. Finally, the extracts present in the acceptor compartment are collected and were ready to be analyzed.

In order to avoid potential carry over, the donor plate was discarded after use and the acceptor plate was rinsed with a mixture of iPrOH/H2O 50:50 (*v*/*v*) and dried under nitrogen after each experiment.

### 2.6. Capillary Electrophoresis–Mass Spectrometry

CE separations were carried out using a G7100 capillary electrophoresis (CE) system from Agilent (Waldbronn, Germany) using a fused silica capillary (BGB Analytik Benelux B.V, Harderwijk, The Netherlands) with a length of 90 cm and an internal diameter of 50 µm. Separation was carried out using a background electrolyte (BGE) composed of 10% acetic acid (*v/v*). Prior to first use, the capillary was conditioned with MeOH, H_2_O, 1 M NaOH, H_2_O, 1 M HCl, H_2_O, 0.1 M HCl, H_2_O, and BGE at 5 bar. Between each run, the capillary was rinsed with MeOH and BGE at 5 bar for 130 s. CE-MS analyses were performed using a MSI-CE-MS approach with electrokinetic plugs between samples which were hydrodynamically injected [20]. Briefly, the first sample was hydrodynamically injected at 100 mbar for 20 s (corresponding to 1.6% of total length of the capillary). Prior to the second sample injection, a voltage of +30 kV was applied during 60 s. This process was repeated until 7 samples were injected. Typically, the first sample injected consisted of the neat standard at 2.5 µg/mL, followed by six injections of other samples consisting of the replicates of a specific extraction condition. The samples were kept at 7–8 °C in the auto-sampler using an external water cooling system.

The CE system was hyphenated with an Agilent 6230 TOF mass spectrometer (Santa Clara, CA, USA) via an electrospray ionization (ESI) source and a coaxial sheath-flow ESI interface equipped with a stainless-steel triple-tube sprayer (P/N G1607A) from Agilent Technologies. The sheath liquid was composed of a mixture of H_2_O/iPrOH/acetic acid 50:50:1 (*v/v/v*) and delivered at a flow rate of 3 µL/min using a 2300 Series isocratic pump (Agilent Technologies) equipped with a 1:100 split ratio. MS experiments were acquired in positive mode between 50 and 1000 *m/z* with an acquisition rate of 1.5 spectrum/s. The nebulizer gas was set to 0 psi, while the sheath gas flow rate and temperature were set at 11 L/min and 100 °C, respectively. The ESI capillary voltage was adjusted to 5500 V. Fragmentor and skimmer voltages were set at 150 V and 50 V, respectively. MassHunter version B.06.00 (Agilent, Santa Clara, CA, USA) was used for data acquisition, instrument control, and data treatment. Isopropanol acetate adduct [CH_3_COOH-C_3_H_8_O+H]^+^ (*m/z* 121.08592) was used as reference mass for TOF calibration of each spectrum.

### 2.7. Fast-Liquid Chromatography–Mass Spectrometry

A previously developed fast LC-MS/MS method for analysis of metabolites linked to gut metabolism was used in this study [10]. Briefly, a 1290 Infinity II ultra-high pressure liquid chromatography (UHPLC) system from Agilent Technologies (Waldbronn, Germany) was used for fast LC-MS/MS experiments. The instrument was equipped with an autosampler, a column oven and a binary pump with a maximum delivery flow rate of 5 mL/min. Separations were performed with a Waters AccQ-TagTM Ultra column (2.1 mm × 100 mm, 1.7 µm) maintained at 60 °C. The mobile phases consisted of 0.1% formic acid (A) and MeCN (B). The injection volume was 1 µL and the flow rate of the mobile phase was set at 0.7 mL/min.

The separation was carried out using the following gradient: (B) maintained at 5% for 0.8 min, further increased to 50% over 0.05 min, followed by an increase to 100% in 0.1 min. These conditions were kept during 1.25 min before returning to initial conditions in 0.02 min and re-equilibration over 0.8 min. The total analysis time was 3 min.

The UHPLC system was hyphenated with an AB Sciex 6500 Q-Trap MS (AB Sciex, Concord, ON, Canada) equipped with a Turbo Spray ionDrive source. MS experiments were performed in the positive ionization mode using the selected reaction monitoring (SRM) acquisition mode. The precursor and product ions that were monitored for each compound, as well as the respective collision energies, are reported in Table S1. The SRM experiments were acquired with a chromatographic time window of 60 s and a cycle time of 0.2 s. The drying gas temperature and flow rate were set at 220 °C and 14 L/min, respectively. The ion source gas 1 and 2 pressures were fixed at 80 and 70 psi, respectively, with a temperature of 350 °C for both.

The ion spray voltage, declustering potential, and collision cell exit potential were adjusted to 2500 V, 70 V, and 10 V, respectively. The curtain and collision gas were set at 20 psi and “medium”, respectively.

Data acquisition and instrument control were monitored using AB Sciex Analyst version 1.6.2 (AB Sciex, Concord ON, Canada). Data treatment was performed using Skyline-daily version 4.1 (MacCoss Lab, Seattle, WA, USA).

### 2.8. Calculation of Extraction Yield, Process Efficiency, and Matrix Effect

The extraction yield (EY) is described as the recovery in absence of matrix. In this study, EY was determined by comparing a neat standard solution (2.5 µg/mL) with a neat spiked solution (2.5 µg/mL in 50 mM FA) extracted with Pa-EME [30], according to Equation (1).

(1) $$EY=\frac{AUC_{extract}}{AUC_{neat\;standard}}\times \frac{V_{donor}}{V_{acceptor}}$$

where AUC_extract_ is the peak area of the compound measured in the acceptor solution, AUC_neat standard_ the peak area of the compound in the neat standard solution, V_donor_ the volume of the donor compartment, and V_acceptor_ the theoretical volume recovered in the acceptor compartment.

Process efficiency (PE) describes the extraction performance in presence of matrix and is determined by comparing a neat standard solution (2.5 µg/mL) to a spiked biological sample (2.5 µg/mL) extracted with Pa-EME [31]. The PE was calculated according to Equation (2).

(2) $$PE=\frac{AUC_{extracted\;plasma}}{AUC_{neat\;standard}}\times \frac{V_{donor}}{V_{acceptor}}$$

where AUC_extracted plasma_ is the peak area of the compound measured in the acceptor solution.

The ME is defined as the difference in signal due to ion suppression or signal enhancement. The ME was evaluated using a method described by Matuszewski et al. [31]. In this case, the sample was first extracted and then spiked with compounds of interest to a known final concentration. This post-extraction spiked sample was then compared to a neat standard at the same concentration.

The ME was calculated according to Equation (3).

(3) $$ME=1-\frac{AUC_{post\;extraction\;spiked\;matrix}}{AUC_{neat\;standard}}$$

where AUC_post extraction spiked matrix_ corresponds to the peak area of compounds detected in a biological matrix spiked after extraction with a known analyte concentration and AUC_neat standard_ the peak area of the compound at known concentration measured in neat standard.

## 3. Results and Discussion

TMAO, choline, betaine, l-carnitine, and deoxy-l-carnitine, known predictive biomarkers for CVD [2], were used as model compounds. All these metabolites are very polar compounds with logP between −4.49 and −0.93 (Table 1). Due to their very low lipophilicity, these highly polar molecules are difficult to extract using conventional sample preparation methods such as solid-phase extraction or reversed-phase SPE [9], which are based on the partition coefficients of analytes in two-phase systems. Indeed, only PP has been reported as efficient sample pre-treatment for these class metabolites so far [31,32]. PP is typically used in metabolomics, especially in large-scale studies where high throughput is essential. Fast analytical techniques are also required, such as fast LC-MS, but may lead to strong ME, typically for poorly-retained compounds and particularly in combination with straightforward sample pre-treatments [9,33]. This ME issue highlights the needs for novel sample preparation approaches adapted to the extraction of highly polar compounds.

### 3.1. Optimization of the Parallel Electromembrane Extraction Set-Up

The EME experimental conditions, i.e., applied voltage and sample composition, were first optimized to reach the highest EY and PE while lowering ME. Because most of the compounds of interest are endogenously present in human plasma, dISTDs were used during the Pa-EME optimization step, except for bupivacaine, which is a xenobiotic compound. Based on previous work [20], 2-nitrophenylpentyl ether (NPPE) as SLM and 1% acetic acid (pH 2.8) as both acceptor and donor solutions were used as starting conditions. NPPE was selected due the expected good extraction recovery and low extraction variability for the selected compounds, while 1% acetic acid allowed for both protonation of the basic moiety and neutralization of carboxylic group of l-carnitine, deoxy-l-carnitine and betaine. Moreover, 1% acetic acid generated a relatively low current which allowed for the application of higher voltages without generating excessive Joule heating. The obtained extracts were then analyzed using MSI-CE-MS. MSI-CE-MS consists of consecutive injections of up to seven different samples within the same analytical run. This leads to a significant increase in analysis throughput as well as decrease of analytical variability for sample injected in the same run [34,35,36].

**Figure 2:**
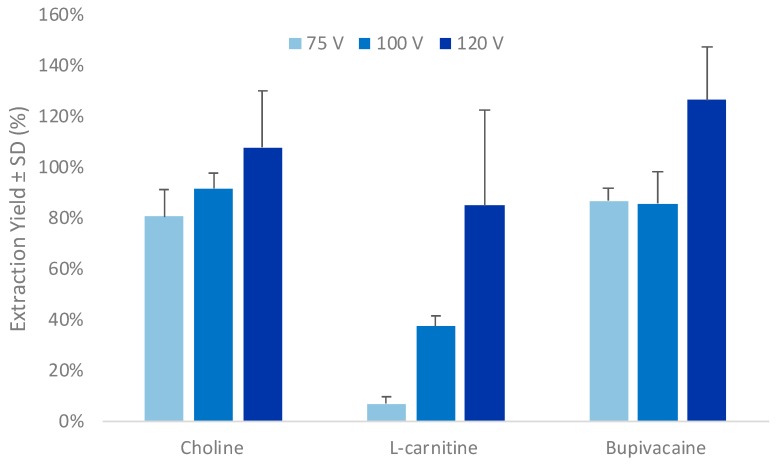
Effect of applied voltage on extraction yield (*n* = 3). Error bars are expressed as the standard deviations.

**Figure 3:**
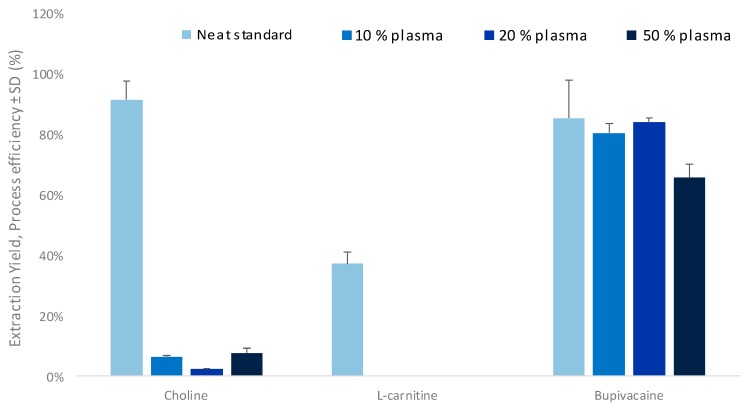
Effect of untreated plasma content in the acceptor compartment on process efficiency (*n* = 3).

First, the extraction voltage was investigated, since EME recoveries are known to be directly correlated to the electric field applied during the electroextraction process [14,29]. Figure 2 and Figure 3 illustrate the results obtained for three compounds, i.e., choline (positively charged and polar), l-carnitine (partially charged and polar), and bupivacaine (positively charged and non-polar). As shown in Figure 2, the EY (calculated according to Equation (1)) increased for all compounds with an increased extraction voltage. The gain in EY was especially important for l-carnitine, where the EY showed a 5-fold enhancement when increasing the voltage from 75 V to 100 V. At 100 V, EYs up to 92% were obtained, with relative standard deviations (RSDs) as low as 4%. Choline and l-carnitine are close compounds with logD values in the same range, i.e., −4.6 and −4.8 at pH 2.8, respectively. The difference observed in EY between both compounds might be explained by the higher molecular charge of l-carnitine and the partial deprotonation of its carboxylic group (pKa 4.2) at pH 2.8, leading to a decrease of its net charge [37,38] and thereby its susceptibility to electromigration. As expected, bupivacaine was easily extracted with EY above 85% with all tested voltages, showing that the Pa-EME setup was functioning well.

The highest voltage tested, i.e., 120 V, led to the highest EYs for all compounds. However, at this voltage, a significant fluid leakage between the acceptor and donor plate was observed. This was explained by gas production caused by electrolysis in the acceptor compartment, leading to an overpressure in this closed compartment and, ultimately, to the loss of the acceptor phase. This supports the apparent higher EY that were observed due to an overestimation of the acceptor compartment volume according to Equation (1).

Therefore, an applied voltage value of 100 V was selected for further experiments, leading to the highest EYs without any volume loss observed.

The influence of the concentration of untreated plasma in the donor compartment on the extraction was then investigated. As shown in Figure 3, a significant drop of PE was observed for highly polar compounds in the presence of 10% untreated plasma or higher. The strong decrease in PE for betaine, l-carnitine, and deoxy-l-carnitine might be explained by partial deprotonation of their carboxylic acid group due to a pH increase in the compartment caused by addition of plasma (up to pH 4.5 with 50% of plasma) and the poor buffer capacity of acetic acid 1%. Therefore, the pH increase of the donor compartment led to a decrease of metabolite net charge. In these conditions, TMAO and choline remained both fully ionized, irrespective of the pH.

The observed decrease in PE for the two polar compounds might be further explained by the drastic reduction of the electric field in the system due to the plasma ionic strength, leading to lower logD values of polar compounds and slower migration into the SLM [39]. On the other hand, bupivacaine was more slightly affected by this phenomenon when using up to 20% of plasma content, due to the known high logD of non-polar compounds in the SLM. However, when using 50% of plasma content, the important increase of ionic strength decreased the electric field, leading to a significantly lower PE for this analyte.

Another hypothesis, i.e., the disturbance of the interface between the organic layer and the plasma sample due to a superficial protein precipitation in this region, seems unlikely since a vortex is created in the donor compartment thanks to the very high agitation rate (i.e., 1400 rpm). In addition, since bupivacaine is a drug known to be 95% linked to plasmatic proteins but showed high PE values, the protein-binding hypothesis was discarded. However, PVDF material is well-known for its very high affinity and protein binding capacity. Therefore, this might lead to perturbation of the organic layer by competition between the organic solvent and proteins.

Finally, as high sensitivity is essential in metabolomics, an untreated plasma content of 10% was selected for further experiments.

In order to test our two hypotheses, we evaluated two approaches to modify the sample composition, namely, (1) addition of an organic solvent to the sample (e.g., MeOH) to enhance the electric field, and (2) PP prior to extraction to remove proteins.

Various proportions of MeOH were added to the donor compartment, i.e., 10%, 20%, and 50%, but no significant difference in PE was observed (data not shown). Higher concentrations of organic modifier were not tested to avoid possible SLM dissolution [40].

**Figure 4:**
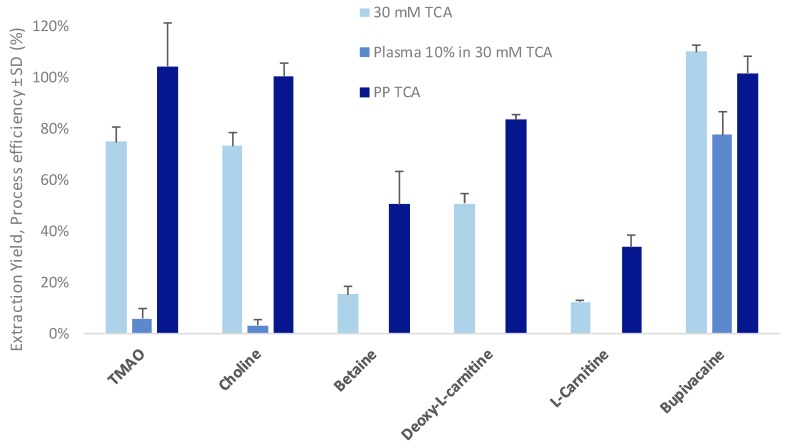
Influence of protein precipitation (PP) on process efficiency (PE) (*n* = 6). Experimental conditions: current, 400 µA/well; extraction time, 15 min; agitation, 1400 rpm, 1% acetic acid as acceptor compartment. TCA: trichloroacetic acid; PP-TCA: protein precipitation using trichloroacetic acid, ratio trichloroacetic acid/sample 0.05:1 (*v/v*).

PP using TCA is known to allow for an efficient protein removal (i.e., above 99%) with a limited dilution factor [41,42]. The PP-TCA method takes place in pure aqueous phase, showing the benefit of avoiding the evaporation step that is necessary when using organic solvents for PP, which is favorable for compatibility with the EME approach. Moreover, the low pH that occurs when using PP-TCA is suitable for the extraction of cationic compounds. Therefore, PP-TCA was selected for further investigations, in a 0.05:1 ratio (TCA:sample). After a 10-fold dilution of the precipitated plasma, the TCA concentration was close to 30 mM, which was sufficient to obtain a pH of 2.0 and ensure protonation of all the compounds of interest. With an applied voltage of 100 V, a high current (more than 1–2 mA/well) was observed. This relatively high current could be explained by higher conductivity of the solution of 30 mM TCA compared to 1% acetic acid. In order to avoid the potential issues generated by a high current, the extraction current was set to 400 µA/well to minimize electrolysis and gas production, while maximizing both EY and PE. As presented in Figure 4, good EY (up to 75% for polar compounds) and low variability (as low as 7%) were obtained using the optimal conditions.

For all the selected compounds, similar or higher PE (up to 100%) and low variability (as low as 2%) were obtained on protein precipitated plasma samples compared to neat solutions, especially for betaine and l-carnitine where PE were 3.3 and 2.7-fold higher, respectively, when PP-TCA was used. This increase in PE remains unexplained and requires further investigations. Nevertheless, a PP step appears essential prior to EME of highly polar compounds to reduce both ionic strength and buffer capacity of biological fluids. To assess the maximal precipitated plasma volume extractable with the developed approach, different plasma contents, i.e., up to 50%, were evaluated. Using the same PP-TCA method, an increase of the precipitated plasma content into the donor compartment involved an increase of TCA concentration, up to 150 mM. The results are summarized in Table 2.

Except for L-carnitine, good PE (between 50% and 100%) values were obtained for all tested precipitated plasma amounts. No change in PE was observed for TMAO, choline, and bupivacaine. An increase of PE for betaine with an increased plasma content and TCA concentration was obtained, explained by the lower pH observed, i.e., a pH value of 1.0–1.5 with 50% of plasma and 150 mM of TCA versus pH of 2.0 with 30 mM of TCA and 10% of plasma content. This decrease of pH induces a higher positive net charge on betaine, leading to an increase of its electrophoretic mobility. Nevertheless, the decrease in PE observed for deoxy-l-carnitine and carnitine remain unexplained but might be the consequence of stability issues of these metabolites in highly acidic conditions (pH ~1).

Finally, the linear response function of the developed method was evaluated. For this purpose, a fast LC-MS/MS method was used to be in similar conditions as what is observed in the context of large cohort studies with thousands of samples. The calibration curve was plotted using ratios of non-deuterated compounds and dISTDs in a neat solution. Calibration samples with increasing concentrations of TMAO, choline, betaine, l-carnitine and deoxy-l-carnitine (Table 2) were made and mixed to constant concentrations of their dISTD in 30 mM TCA, to mimic EME conditions of the donor compartment composition. These calibration samples were extracted using the optimized Pa-EME setup and the selected experimental parameters (i.e., 400 µA/well, 15 min, 1400 rpm). As shown in Table 2, a linear response function (R^2^ > 0.995) was obtained on concentration ranges of two orders of magnitude for all compounds of interest (Figure S1).

### 3.2. Evaluation of Matrix Effects

The ME were evaluated for the developed Pa-EME set-up in combination with fast-LC-MS/MS.

**Figure 5:**
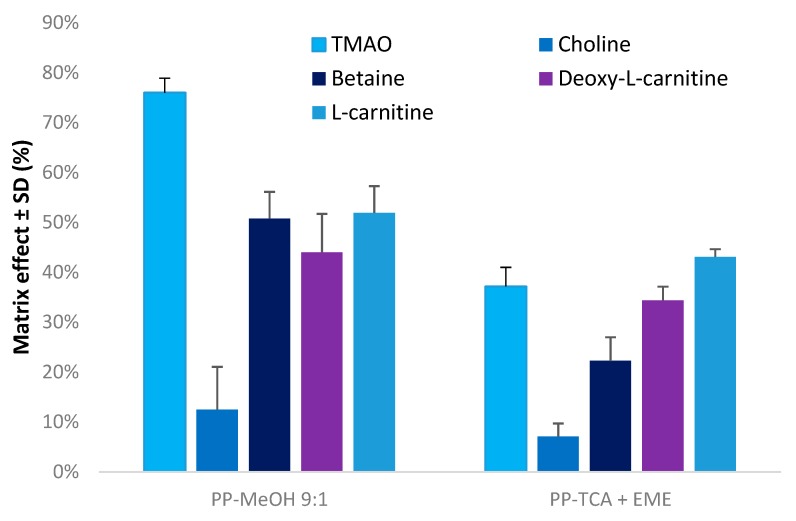
Comparison of matrix effect between conventional PP-MeOH and combination of PP-TCA and Pa-EME using optimal conditions. PP-MeOH: protein precipitation using methanol, ratio methanol/sample 9:1 (*v/v*).

The observed ME when using a conventional PP-MeOH were compared to the method combining PP-TCA and Pa-EME. For all these experiments, the same amount of untreated plasma concentration was used, i.e., 10 µL of untreated plasma for 100 µL of precipitated plasma for PP-MeOH and 30 µL of untreated plasma for 300 µL of donor solution for EME, respectively, leading in both case to an equivalent of 10% of untreated plasma after PP. As shown in Figure 5, lower MEs were observed using the combination of PP-TCA and Pa-EME. Compared to the conventional PP-MeOH approach, a noticeable decreased ME was observed, especially in the case of TMAO and betaine where the observed ME was 2-fold lower with the combined approach versus MeOH-PP alone. This decrease might be explained by the cationic selectivity of EME and efficient salt removal.

Along with the decrease in ME and high PE, a 2.9-fold increase of peak intensity was observed with EME compared to PP-MeOH for TMAO. The sensitivity observed for choline and deoxy-l-carnitine was not significantly impacted, with an increased factor of 1.2 and 1.1, respectively. With same signal intensity, the lower ME observed for betaine compared to PP-MEOH compensated for the low PE of this compound (i.e., 55%). Finally, l-carnitine, which was the compound with the lower PE (i.e., 34%), showed reduced ME but was detected with a signal intensity 0.6-fold lower than with PP-MeOH.

### 3.3. Application to Metabolomics Studies

The potential of the developed Pa-EME set-up for large scale metabolomics studies has been investigated by correlating the data obtained with this optimized extraction method (combination of PP-TCA+EME) versus a typically used sample clean-up, i.e., PP-MeOH, on a set of 40 human plasma samples.

Due to limited volume of plasma available (ca. 25 µL), the developed PP-TCA was first downscaled. This adjusted PP-TCA method led to an equivalent of 5% of untreated plasma after PP. The downscaled method led to similar extraction performance compared to the conventional PP-TCA (data not shown), and was therefore used for subsequent experiments.

**Figure 6:**
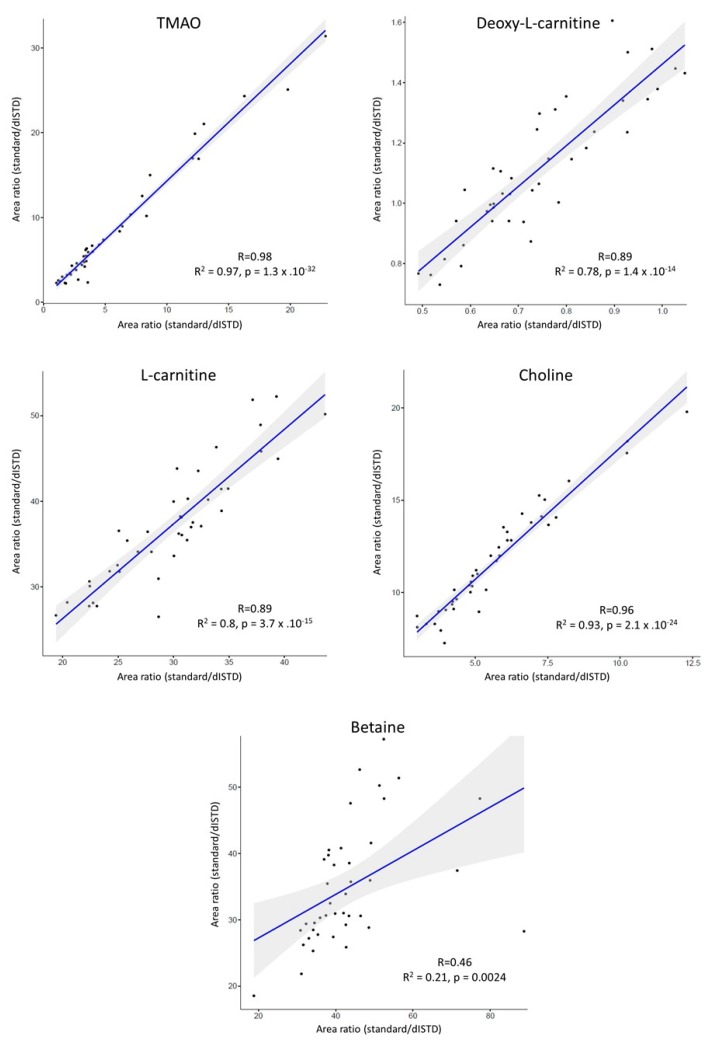
Area ratio correlations observed for analyte response between PP-TCA + EME (*x*-axis) and PP-MeOH (*y*-axis). 95 % confidence intervals were calculated using Pearson correlation. dISTD: deuterated internal standard.

As shown in Figure 6, excellent correlations were obtained between the two evaluated sample preparation approaches for TMAO, choline, l-carnitine, and deoxy-l-carnitine, with correlation coefficients (R) between 0.88 and 0.98. In addition, the linear models are highly significant, with *p*-values between 1.4 × 10^−14^ and 1.3 × 10^−32^. These results highlight the relevance of the developed Pa-EME method in comparison with gold standard methods used for sample preparation in metabolomics. However, a poor correlation was observed for betaine (R = 0.46), probably due to a contamination of the milliQ water used for preparation of acceptor and donor solution with a compound detected in the same SRM transition as betaine.

During the analysis of these clinical samples, an unexpected boiling and loss of acceptor compartment was observed for many samples during the EME process. This unexpected phenomenon was likely explained by the differences in plasma composition. Indeed, the optimization phase was carried out using the same sample split in six aliquots which were simultaneously extracted using Pa-EME. Therefore, the total applied current (2.4 mA) was equally distributed over the six wells, leading to a current of 400 µA/well. No boiling or loss of acceptor phase was observed during the optimization process. However, with a parallel extraction of six different plasma samples, the applied current was not uniformly distributed over the wells. Indeed, due to small differences of sample composition (e.g., ionic strength, residual proteins, etc.), plasma samples possess different conductivities. According to Kirchhoff’s current law, these different conductivities lead to different current in every parallel circuit. Consequently, several samples were subjected to currents lower than 400 µA and other samples to a current above this limit, the latter leading to boiling and loss of acceptor volume. However, this variability was successfully corrected using a dISTD for each compounds of interest, as shown by the good correlation obtained with PP-MeOH (Figure 6). A solution to circumvent this issue could be to use lower currents to stay below the limit of 400 µA/well, but this solution requires enhanced extraction times or would lead to lower PE.

## 4. Conclusions

This study demonstrated the power of EME approaches for the extraction of highly polar compounds from biological fluids and its potential for metabolomics studies. In this study, we presented an optimized Pa-EME method for the efficient extraction of highly polar compounds from plasma. The developed Pa-EME method involves a PP step using TCA before the actual EME process; this is an essential step to reduce the buffer capacity of plasma, and additionally to avoid possible interferences with the PVDF membrane. We demonstrated that the combination of PP-TCA with Pa-EME allowed for high PE (up to 100%) as well as low variabilities (RSD as low as 7%) for the extraction of selected highly polar compounds from plasma samples. Moreover, the PP-TCA-Pa-EME set-up led to decreased ME in comparison to conventional PP-MeOH when using fast chromatography (up to 2-fold matrix effect reduction for TMAO and betaine). A 3-fold gain in sensitivity was observed for TMAO with PP-TCA-Pa-EME compared to PP-MeOH. Similar sensitivity was obtained for choline, betaine, and deoxy-l-carnitine. However, using PP-TCA-PaEME, l-carnitine presented a decrease of sensitivity in comparison with PP-MeOH due to incomplete extraction. This poor sensitivity could be nevertheless improved with further experiments and the development of a new Pa-EME set-up for higher enrichment with favorable acceptor/donor volume ratio. The developed method showed a linear response function (R^2^ between 0.994 and 0.997) of more than two orders of concentration magnitude for all the metabolites of interest.

Finally, the developed PP-TCA-Pa-EME method was compared to PP-MeOH using 40 different plasma samples. The influence of sample conductivity, which is a common concern in electromigration-based sample pre-treatment, was highlighted but was fully compensated using dISTDs for each compound. Overall, the combination of PP-TCA and EME showed excellent correlation with the conventional PP-MeOH.

The great potential of electromembrane extraction in bioanalysis is highlighted by its analytical merit in terms of high recovery (up to 100%) and low variability (down to 7%) of highly polar metabolites from a complex matrix such as plasma, a significant reduction of the matrix effect, and the strong correlation to gold standard sample preparation practices in metabolomics.

The development of a new Pa-EME device to further reach higher enrichment factors for such metabolites will represent the next logical step for application of this method to state-of-the-art metabolomics-based analysis.

## Figures and Tables

**Table 1:** Physicochemical properties of the compounds of interest.

	Molecular Weight (Da)	LogP *	pKa *	Structure	ChEBI
**TMAO**	75.11	−0.9	4.7	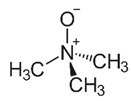	15724
**Choline**	104.17	−4.7	14.1	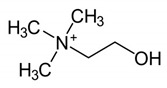	15354
**Betaine**	117.15	−4.5	2.3	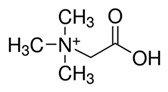	17750
**Deoxy-l-carnitine(4-trimethylammoniobutanoic acid)**	145.20	−4.0	4.5	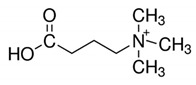	16244
**l** **-carnitine**	161.20	−4.9	4.2	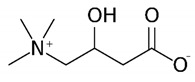	17126
**Bupivacaine**	288.43	4.5	13.6	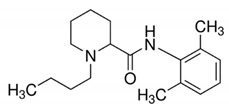	3215

*: calculated using Chemaxon, www.chemicalize.org. TMAO: trimethylamine N-oxide.

**Table 2:** PE (RSD) in % (*n* = 6) according to the precipitated plasma amount in the sample using the PP-TCA method and linearity ranges obtained using 30 mM TCA in water. RSD: relative standard deviations.

	PE (RSD) in %			Dynamic Range (µM)	R^2^
	10% Plasma 30 mM TCA	20% Plasma 60 mM TCA	50% Plasma 150 mM TCA		
**TMAO**	104 (16)	96 (13)	94 (13)	0.27–43.19	0.997
**Choline**	100 (5)	84 (4)	89 (4)	1.8–286.5	0.996
**Betaine**	55 (12)	66 (14)	71 (9)	4.6–716.1	0.994
**Deoxy-l-carnitine**	84 (2)	60 (14)	60 (10)	0.1–17.6	0.997
**l-carnitine**	34 (14)	18 (54)	12 (6)	3.5–556.5	0.995
**Bupivacaine**	102 (7)	101 (3)	105 (3)	n.d.	

## Supplementary Materials

The following are available online at https://www.mdpi.com/2218-1989/10/1/4/s1. Table S1: SRM transitions and collision energies used in the developed Fast-LC-MS/MS method. Figure S1: Calibration curves built for TMAO, choline, betaine, l-carnitine and deoxy-l-carnitine in water.

## Abbreviations

CVD	cardiovascular disease
CE-MS	capillary electrophoresis–mass spectrometry
dISTD	deuterated internal standard
EME	electromembrane extraction
EY	extraction yield
FA	formic acid
Fast-LC-MS/MS	fast liquid chromatography–tandem mass spectrometry
HILIC	hydrophilic interaction chromatography
ME	matrix effect
MSI-CE-MS	multisegment injection–capillary electrophoresis–mass spectrometry
NPPE	2-nitrophenylpentyl ether
MeCN	acetonitrile
MeOH	methanol
Pa-EME	parallel electromembrane extraction
PE	process efficiency
PEEK	polyether ether ketone
PP	protein precipitation
PP-MeOH	protein precipitation using methanol, ratio methanol/sample 9:1 (*v/v*)
PP-TCA	protein precipitation using trichloroacetic acid, ratio trichloroacetic acid/sample 0.05:1 (*v/v*)
PVDF	polyvinyldifluoride
SLM	supported liquid membrane
SRM	selected reaction monitoring
TCA	trichloroacetic acid
TMAO	trimethylamine N-oxide
UHPLC	ultra-high pressure liquid chromatography
