# Supplementary material for: Electromembrane Extraction of Highly Polar Compounds: Analysis of Cardiovascular Biomarkers in Plasma
Source: Metabolites. 2019 Dec 18;10(1):4. doi: 10.3390/metabo10010004 (PMC7022788; doi:10.3390/metabo10010004)
Supplement: Supplementary file 1 [file metabolites-10-00004-s001.zip › metabolites-656659-Supplementary Materials/Figure S2.pdf]

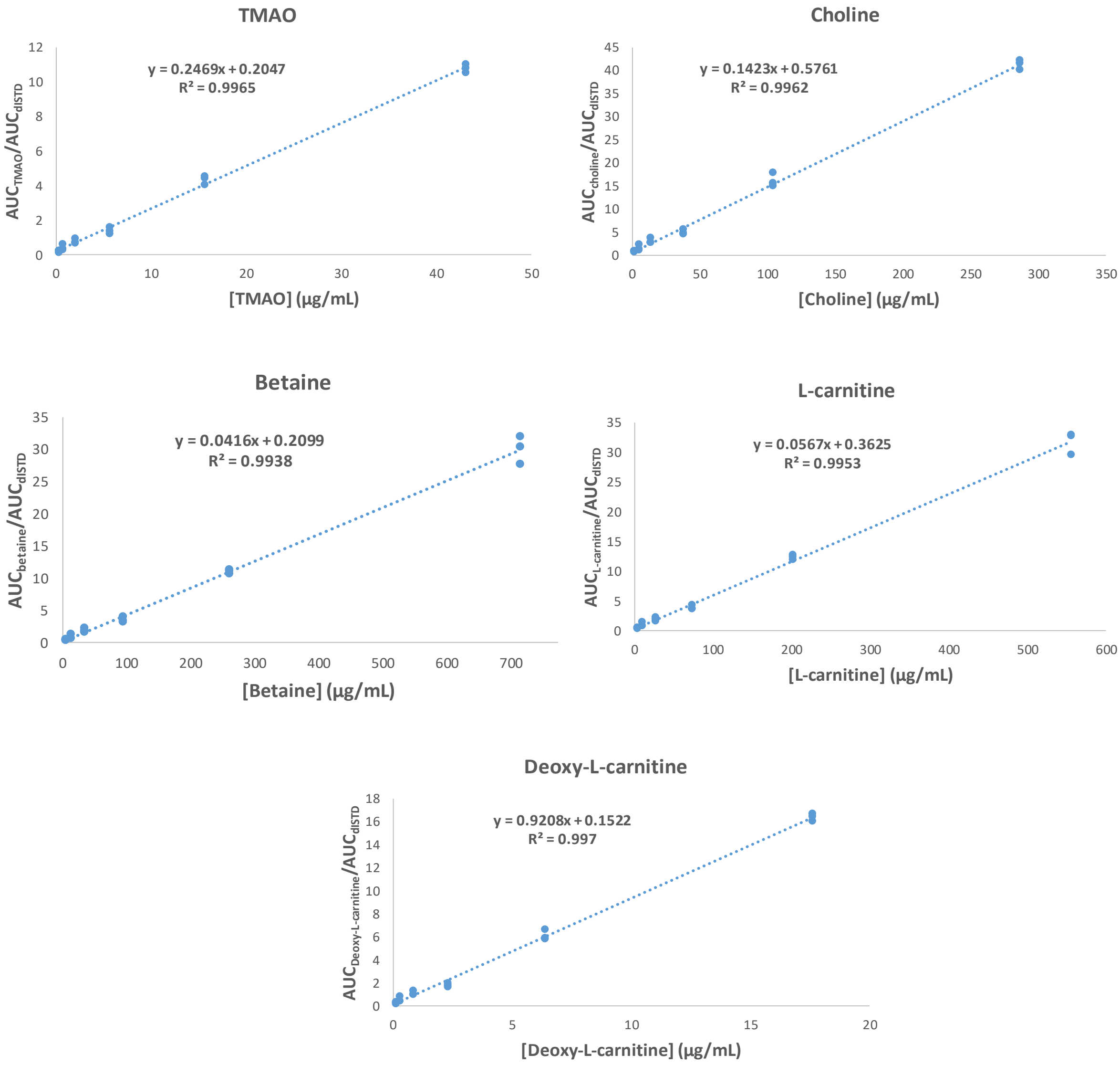

Figure S.2: Calibration curves obtained for TMAO, choline, betaine, L-carnitine and deoxy-L-carnitine
