# Supplementary material for: Electromembrane Extraction of Highly Polar Compounds: Analysis of Cardiovascular Biomarkers in Plasma
Source: Metabolites. 2019 Dec 18;10(1):4. doi: 10.3390/metabo10010004 (PMC7022788; doi:10.3390/metabo10010004)
Supplement: Supplementary file 1 [file metabolites-10-00004-s001.zip › metabolites-656659-Supplementary Materials/Table S1.pdf]

Table S.1: Selected reaction monitoring transitions and collision energies used in the Fast-LC-MS/MS platform

Compounds	Precursor ion	Product ion	Collision energy
**Betaine**	118.1	58.2	30 eV
**Betaine-d_9**	127.1	66.1	
**L-Carnitine**	162.3	85.1	25 eV
**L-Carnitine-d_3**	165.3	85.1	
**Choline**	104.2	60.1	25 eV
**Choline-d_4**	108.2	60.1	
**DeoxyL-carnitine**	146.2	87.1	20 eV
**Deoxy-L-carnitine-d_9**	155.2	87.1	
**TMAO**	76.1	58.1	25 eV
**TMAO-d_9**	85.1	66.1	
